# Sex gap in aging and longevity: can sex chromosomes play a role?

**DOI:** 10.1186/s13293-018-0181-y

**Published:** 2018-07-17

**Authors:** Gabriel A.B. Marais, Jean-Michel Gaillard, Cristina Vieira, Ingrid Plotton, Damien Sanlaville, François Gueyffier, Jean-Francois Lemaitre

**Affiliations:** 1 Laboratoire “Biométrie et Biologie Evolutive”- UMR 5558, CNRS / Université Lyon 1, Villeurbanne, France; 2 Service d’Endocrinologie Moléculaire et Maladies Rares, Hospices Civils de Lyon, Lyon, France; 3 Service de Génétique, Hospices Civils de Lyon, CRNL, GENDEV team, INSERM U1028, CNRS UMR5292, Université Lyon 1, Lyon, France

**Keywords:** Longevity, Aging, Sexual dimorphism, Sex hormones, Mother’s curse, Sex chromosomes, Transposable elements, Turner, Klinefelter

## Abstract

It is well known that women live longer than men. This gap is observed in most human populations and can even reach 10–15 years. In addition, most of the known super centenarians (i.e., humans who lived for > 110 years) are women. The differences in life expectancy between men and women are often attributed to cultural differences in common thinking. However, sex hormones seem to influence differences in the prevalence of diseases, in the magnitude of aging, and in the longevity between men and women. Moreover, far from being human specific, the sex gap in longevity is extremely common in non-human animals, especially in mammals. Biological factors clearly contribute to such a sex gap in aging and longevity. Different hypotheses have been proposed to explain why males and females age and die differently. The cost of sexual selection and sexual dimorphism has long been considered the best explanation for the observed sex gap in aging/longevity. However, the way mitochondria are transmitted (i.e., through females in most species) could have an effect, called the mother’s curse. Recent data suggest that sex chromosomes may also contribute to the sex gap in aging/longevity through several potential mechanisms, including the unguarded X/Z, the toxic Y/W and the loss of Y/W. We discuss future research directions to test these ideas.

## Background

Global Health Observatory data shows that global life expectancy at birth in 2015 was 73.8 years for women and 69.1 years for men [1]. This “sex gap” in survival prospects is found in nearly all countries where longevity records are officially compiled [2]. When comparing the sex distribution of very old age classes, women are generally over-represented [3]. For instance, a detailed analysis of the Calabrian (southern Italy) population revealed that there are twice as many women as men in the living centenarians [4], a sex bias that is also observed among super-centenarians (i.e., older than 110 years, see e.g., [5]). The over-representation of women in the very late age classes has been observed for a long time [6], and it is therefore not a surprise that the current longevity record for humans (i.e., 122 years of age) belongs to a woman, Jeanne Calment (1875–1997; [7]).

**Fig. 1:**
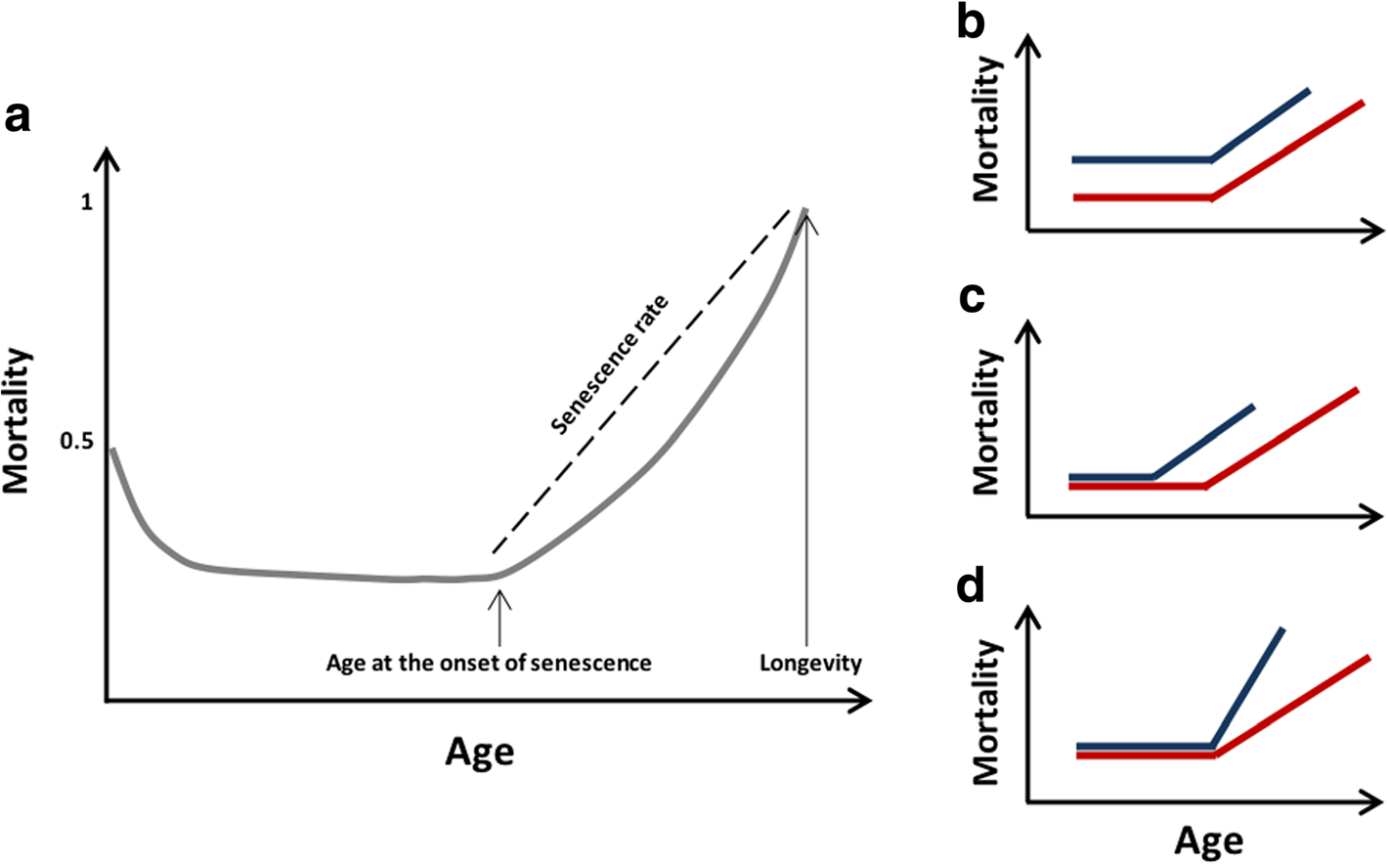
Graphical display of some mortality metrics mentioned in the article. **a** represents a standard age-specific mortality curve for a mammalian species. Mortality decreases from birth to early adulthood, then stays low and relatively constant (i.e., prime-age stage) and finally starts to increase. The age when mortality starts to increase is the age at the onset of senescence or aging, and the intensity of the increase in the mortality rate with age is defined by the rate of senescence or aging. Males and females can differ in longevity in various ways. For instance, males and females can differ in the annual adult mortality (**b**), the age at the onset of aging and (**c**) the rate of aging (**d**). We did not represent scenarios where more than one trait can differ between males and females (see Box 1 for a thorough definition of the mortality key terms)

Sex differences in lifespan were even labeled ‘*one of the most robust features of human biology*’ ([2], p1026) because this female survival advantage has been observed since sex-specific longevity data are recorded (i.e., the middle of the eighteenth century in some countries, [3, 8]), although a progressive reduction of the sex gap is being observed in some ‘low mortality’ countries (sensu [9]). The direction of the sex gap in lifespan (or longevity, see Box 1, Fig. 1) is the same in almost all populations or countries. The magnitude of the sex-difference, however, varies across populations [8, 10, 11]. Rochelle and colleagues have recently emphasized this point by comparing male and female life expectancy at birth (see Box 1) across 54 countries worldwide. In all 54 countries analyzed, life expectancy at birth was higher in females than in males, with a mean female advantage of 5.8 years. Nevertheless, the sex-gap in life expectancy at birth varied from 1 to 14 years according to the country considered [11].

This higher survival observed in women has attracted much attention from the biomedical world [2, 12–16]. Many diseases (but not all) affect more men than women. For the two main causes of death in the USA, heart disease and cancer, the mortality rate is higher in men than in women at a given age [15]. Sex hormones are considered key in explaining these patterns [15, 16]. Estrogen is thought to have a protective role against many diseases, while testosterone may increase the risk of developing diseases and reduce lifespan [15, 16]. Consistent with this idea, the chances of suffering from hypertension and developing Alzheimer’s disease, two important causes of death for women, greatly increase in case of decreased estrogen production, either natural (menopause) or from surgical cause [15, 17] (but see next paragraph). For some diseases, however, estrogen and late menopause may increase risks, as in breast cancer risks, for example [15]. Although the mechanistic details of how sex hormones could affect aging and longevity are far from being understood, the association between sex hormones and diseases makes sense as estrogen and testosterone are known to have quite different effects on many aspects of human development and physiology [15, 16].

However, clinical trials using sex hormones have been disappointing. A randomized clinical trial tested specifically whether reproducing estrogen impregnation in men decreased the risk of recurrent myocardial infarction [18]. The trial was prematurely stopped because of a marked 47% increase in the rate of myocardial infarction compared to that of the placebo. Randomized controlled trials conducted in women after cardiovascular accidents tested whether artificially prolonging estrogen impregnation after menopause maintained the benefit of women over men in terms of the cardiovascular risk. Here, again, the trials did not confirm the expected benefit [19], whereas observational studies advocated it [20]. Estrogen has some antioxidant and anti-inflammatory properties [21] and the JUPITER trial showed that a statin drug opposed to the placebo reduces the risk of myocardial infarction through cholesterol control but also through decreasing C-reactive protein, a common marker of inflammation [22], in individuals specifically recruited on the high-normal values of this marker. The association between sex hormones and diseases might thus be a very indirect one.

Studies on castrated males (eunuchs, mentally ill institutionalized men) suggest that reduced testosterone production is associated with an increase (of ~ 10–15 years) in life expectancy [23, 24] (but see [25]). However, the explanation is not clear. It could be that reduced testosterone production is not harmful for the body. It could also be that less testosterone reduces high-risk behaviors (e.g., aggressive behaviors, drug abuse, [26]). Another study comparing monks and nuns found similar life expectancies in both cloistered (protected) populations during 1870–2000 in Germany [27], which supports the “high-risk behaviour” hypothesis. Interestingly, less protection in the monk lifestyle since the 70’s was associated with monks dying more of high-risk behaviors [28]. However, monks and nuns did not experience completely similar environments. Nuns used to work in hospitals and were more exposed to infectious diseases compared to monks, probably decreasing their life expectancy, which may have masked the potential harmful effects of testosterone for the body in monks [27, 29].

The differences in the life expectancy between men and women are often attributed to cultural differences in common thinking. Indeed, several studies have highlighted the importance of social factors (e.g., smoking, alcohol consumption, poor social status) in the variation in the sex gap in longevity among human populations (e.g., [11, 29, 30] and references therein). However, the association between sex hormones to sex-specific patterns of disease, aging and longevity mentioned above suggests that biological factors are also at work. Another line of evidence supports a strong role of biology: females outlive males in most mammals, not just in humans, as we will discuss in the next section.

## Sex gap in aging and longevity in non-human animal species

In most animals, males and females show marked differences in their mortality patterns. Whether females outlive males or vice versa differs among taxa. In this section, we present a brief overview of the sex differences in aging and longevity patterns across the tree of life by focusing on the most studied species in that context (i.e., mammals, birds and insects) and in most cases, reviewing studies performed in wild populations.

### Mammals and birds

In non-human mammals, sex gaps in longevity have for a long time been measured through comparisons between the maximum longevity of males and females (see Box 1). Similarly, to what we described for human populations, longevity records are in most species held by females although in some species the sex gap is tiny with no obvious differences (Table 1). Again, as observed in human populations, the direction of the sex gap in lifespan appears to be shared among populations of the same species [31], although with different magnitudes. Different magnitudes of the sex gap among populations of a given species indicate that environmental conditions modulate the general picture, as reported in humans. For instance, when comparing three north-American free-ranging populations of bighorn sheep (*Ovis canadensis*), the sex gap in maximum longevity can change from an almost equal longevity between sexes in some populations, with females outliving males by 5 or 6 years in other populations [32].

**Table 1:** Lifespan and rate of aging comparisons between females and males in mammals, birds or insects

			Lifespan		Rate of aging		References
			Female	Male	Female	Male	
Mammals							
Primates	Sifaka	*Propithecus verreauxi*	**24**	20	0.0991	**0.186**	[118]^a,b^
	Northern Muriqui	*Brachyteles hypoxanthus*	27	27	0.129	**0.148**	[118]^a,b^
	Capuchin	*Cebus capucinus*	**20**	13	0.165	**0.294**	[118]^a,b^
	Yellow Baboon	*Papio cynocephalus*	**28**	23	0.123	**0.213**	[118]^a,b^
	Chimpanzee	*Pan troglodytes*	39	**41**	0.0992	**0.137**	[118]^a,b^
	Gorilla	*Gorilla beringei*	**39**	35	0.211	**0.182**	[118]^a,b^
	Mandrill	*Mandrillus sphinx*	**22.23**	12.70	–	–	[35]^d^
	Rhesus macaque	*Macaca mulatta*	**9.44**	5.51	–	–	[35]^d^
	Japanese macaque	*Macaca fuscata*	**7.33**	4.30	–	–	[35]^d^
	Gelada	*Theropithecus gelada*	**9.62**	7.60	–	–	[35]^d^
Carnivora	Ringed seal	*Phoca hispida*	37	**40**	0.056	**0.057**	[119]^a,c^
	African wild dog	*Lycaon pictus*	3.29	**3.66**	–	–	[35]^d^
	Southern elephant seal	*Mirounga leonina*	**4.18**	3.12	–	–	[35]^d^
Rodentia	Black-tailed prairie dog	*Cynomys ludovicianus*	**2.80**	1.74	–	–	[35]^d^
	North American beaver	*Castor canadensis*	**2.93**	2.75	–	–	[35]^d^
Perrisodactyla	Burchell’s zebra	*Equus burchelli*	49	**55**	**0.106**	0.094	[119]^a,c^
Artiodactyla	Impala	*Aepyceros melampus*	6	6	–	–	[37]^e^
	Gaur	*Bos gaurus*	**11**	6	–	–	[37]^e^
	Wild goat	*Capra aegagrus*	**3.75**	2.5	–	–	[37]^e^
	Alpine ibex	*Capra ibex*	12	**12.5**	–	–	[37]^e^
	Iberian ibex	*Capra pyrenaica*	**6**	5	–	–	[37]^e^
	Wildebeeste	*Connochaetes taurinus*	**5.5**	5	–	–	[37]^e^
	Topi	*Damaliscus lunatus*	3.5	3.5	–	–	[37]^e^
	Topi	*Damaliscus lunatus*	6	6	**0.326**	0.311	[119]^a,c^
	Defassa Waterbuck	*Kobus defassa*	8	8	–	–	[37]^e^
	Lechwe	*Kobus leche*	6	6	–	–	[37]^e^
	Mountain goat	*Oreamnos americanus*	**9**	4	–	–	[37]^e^
	Mountain goat	*Oreamnos americanus*	**8.18**	5.82	–	–	[35]^d^
	Soay sheep	*Ovis aries*	2	2	–	–	[37]^e^
	Soay sheep	*Ovis aries*	**5.13**	3.12	–	–	[35]^d^
	Bighorn sheep	*Ovis canadensis*	**7.5**	5	–	–	[37]^e^
	Dall Mountain Sheep	*Ovis dalli*	9.5	**11**	–	–	[37]^e^
	Dall Mountain Sheep	*Ovis dalli*	13	13	0.118	**0.170**	[119]^a,c^
	Isard	*Rupicapra pyre ica*	11	**12**	–	–	[37]^e^
	Chamois	*Rupicapra rupicapra*	**7**	6.5	–	–	[37]^e^
	African buffalo	*Syncerus caffer*	11	11	–	–	[37]^e^
	African buffalo	*Syncerus caffer*	16	**22**	**0.143**	0.084	[119]^a,c^
	African buffalo	*Syncerus caffer*	7.36	**7.38**	–	–	[35]^d^
	Greater kudu	*Tragelaphus strepsiceros*	**8**	4	–	–	[37]^e^
	Moose	*Alces alces*	**7**	3.5	–	–	[37]^e^
	Roe deer	*Capreolus capreolus*	**8.25**	5	–	–	[37]^e^
	Roe deer	*Capreolus capreolus*	**7.91**	5.01	–	–	[35]^d^
	Elk	*Cervus canadensis*	**18**	8	–	–	[37]^e^
	Elk	*Cervus elaphus canadensis*	**16.05**	7.64	–	–	[35]^d^
	Red deer	*Cervus elaphus*	**10**	7	–	–	[37]^e^
	Red deer	*Cervus elaphus*	**10.6**	8.0	–	–	[35]^d^
	Sika deer	*Cervus nippon*	**11**	8	–	–	[37]^e^
	Black-tailed deer	*Odocoileus hemionus columbianus*	**5**	3	–	–	[37]^e^
	Reindeer	*Rangifer tarandus*	**6.5**	4.5	–	–	[37]^e^
	Reindeer	*Rangifer tarandus*	**16**	11	0.111	**0.212**	[119]^a,c^
	Reindeer	*Rangifer tarandus*	**4.63**	2.18	–	–	[35]^d^
Cetacea	Common bottlenose dolphin	*Tursiops truncatus*	**12.34**	9.74	–	–	[35]^d^
Birds							
Accipitriformes	Osprey	*Pandion haliaetus*	**6.59**	6.48	–	–	[35]^d^
Anseriformes	Tundra swan	*Cygnus columbianus*	27	**32**	**0.101**	0.079	[119]^a,b^
	Tundra swan	*Cygnus columbianus*	5.60	**6.94**	–	–	[35]^d^
	Barnacle goose	*Branta leucopsis*	7.00	**7.80**	–	–	
Ciconiiformes	Black-legged kittiwake	*Rissa tridactyla*	> 12	> 12	**0.074**	0.069	[119]^a,b^
	Black-legged kittiwake	*Rissa tridactyla*	**6.56**	5.24	–	–	[35]^d^
Passeriformes	European pied flycatcher	*Ficedula hypoleuca*	7	7	0.235	**0.241**	[119]^a,b^
	Great tit	*Parus major*	7	7	0.233	**0.242**	[119]^a,b^
	Arabian babbler	*Tursoides squamiceps*	6	**7**	**0.225**	0.211	[119]^a,b^
	Arabian babbler	*Turdoides squamiceps*	2.95	**4.30**	–	–	[35]^d^
	Florida scrub jay	*Aphelocoma caerulescens*	**4.82**	4.52	–	–	[35]^d^
Galliformes	Black grouse	*Tetrao tetrix*	**3.81**	2.53	–	–	[35]^d^
Piciformes	Acorn woodpecker	*Melanerpes formicivorus*	3.08	**4.30**	–	–	[35]^d^
Insects							
Lepidoptera	Edith’s checkerspot	*Euphydryas editha*	–	–	2.0208	**2.7086**	[120]^c^
	Japanese luehdorfia	*Luehdorfia japonica*	–	–	1.8337	**1.8556**	[120]^c^
	Myrtil	*Maniola jurtina*	–	–	0.2994	**1.4000**	[120]^b,c^
	Japanese clouded Apollo	*Parnassius glacialis*	–	–	**3.2305**	2.2751	[120]^c^
	Mormon Fritillary	*Speyeria mormonia*	–	–	1.6364	**1.6837**	[120]^c^
Odonata	Small red damselfly	*Ceriagrion tenellum*	–	–	**0.065**	0.045	[121]^b^
	Bluets	*Enallagma hageni*	–	–	**0.153**	0.143	[121]^b^
	Azure damselfly	*Coenagrion puella*	–	–	0.088	**0.111**	[121]^b^

We limited our literature search to comparative studies including sex-specific lifespan or rate of aging estimates in natural populations of mammals, birds or insects. In bold, the sex with the longest lifespan or the steepest rate of aging

^a^ Lifespan measured as maximum longevity

^b^ Rate of aging measured as Gompertz rate of aging

^c^ Rate of aging measured as Weibull rate of aging

^d^ Lifespan measured as life expectancy at reaching adulthood

^e^ Lifespan measured as the age when 50% of a cohort was still alive

In vertebrates, the increasing number of long-term longitudinal studies since the 1960s, which involve individuals monitored from birth to death [33], now allow getting reliable estimates of age-specific survival in both sexes for many wild populations. The availability of such high-quality data makes the detailed study of sex differences in mortality possible by estimating more accurate metrics of aging (such as the age at the onset of aging and the rate of aging, see Table 1) than the rather crudely observed maximum longevity (see [34] for a thorough discussion on aging metrics in mammals). Not surprisingly, in a wide range of mammalian orders, males generally show a higher rate of aging than females [35–37] even if in some populations the sex gap in aging patterns is not detected (e.g., [38, 39]). Such striking exceptions constitute valuable biological models to better understand the evolutionary roots of the sex gap in aging patterns. In terms of the sex gap in mortality patterns, birds seem to present a much less constant pattern than mammals. In many avian species, differences in age-dependent mortality trajectories are rather tenuous (Table 1). However, contrary to mammals, large-scale comparisons of the sex gap in aging patterns are still lacking in birds. Indeed, to date, most studies that investigated the sex gap in mortality patterns were based on mean adult mortality ([40, 41], see Table 1).

### Insects

Contrary to vertebrates, most insect studies on the sex gap in aging have been performed using laboratory-controlled experiments. Although differences in mating treatments and genotypes can make the sex gap in lifespan extremely variable in laboratory-based experiments [2, 42], it is advantageous to study a tremendous number of individuals, which sometimes allow complex sex-specific mortality trajectories to be revealed. In a demographic study involving approximately 600,000 medflies (*Ceratitis capitata*) of each sex, Carey et al. (1995) showed that mortality is higher in females in early life, then in adults, mortality becomes higher in males between 20 and approximately 60 days of age (i.e., mortality crossover), and finally, mortality does not differ between the sexes later in life [43]. In natural populations of insects, the comparison of survival-related traits between males and females has been mainly done in Diptera (*Telostylinus angusticollis*), where individuals can be marked shortly after emergence. Free-living males appear to live a dramatically shorter life than females (i.e., maximum lifespan of 18 days for females vs. only 10 days for males) and, even more unexpectedly, aging (measured as the increase rate of mortality with age, see Box 1) was only observed in males [44]. A parallel sex-specific comparison was performed on a laboratory-based population of *T. angusticollis* (derived from the same natural population) revealed that females also outlive males in controlled conditions even if the sex gap was much less pronounced [44], which suggests that laboratory assessment of sex differences in mortality might sometimes be misleading for extrapolating what is going on in the wild. Sex differences in lifespan have also been studied in Odonates, since it is possible to mark and monitor individuals in species such as damselflies [45]. However, in this taxon, the sex gap in the lifespan differs across species (Table 1). Overall, it is important to note that insects encompass a much wider set of species than mammals and birds combined, and the sex gap in aging/longevity has been studied so far in very few insect species. This prevents us from drawing any firm conclusions on the overall direction and magnitude of the sex gap in aging/longevity in this taxon.

In the two next sections, we discuss the hypotheses that have been proposed to explain these patterns.

## Sex gap in aging and longevity as a side-effect of sexual selection, sexual dimorphism and sexual conflict

Georges C. Williams (1957) was probably the first to propose a theoretical framework to explain the sex gap in aging [46]. His hypothesis relies on the importance of environmentally driven adult mortality in shaping aging trajectories and more specifically on the fact that a high level of environmentally driven adult mortality should be associated with a shortened lifespan and an increased rate of aging [46, 47]. In the context of sex differences in the mortality trajectories, the sex that is more prone to a high level of environmentally driven adult mortality should thus live a shorter time and exhibit a faster rate of aging [46]. However, increasing evidence that environmentally driven adult mortality might be condition-dependent rather than random has challenged this long-standing idea (e.g., [48]). Indeed, certain sources of mortality are more likely to remove a specific type of individuals from the population. For instance, a pathogen in a population might be responsible for the death of ‘poor quality’ individuals while ‘high quality’ individuals will better cope with this infectious agent and will thus survive and reproduce. In contrast, a tsunami is likely to kill individuals independent of their ‘quality’. High levels of environmentally driven adult mortality can thus be associated with either a reduced or a longer longevity according to complex interactions between the organism, the environmental conditions and the main source of mortality. Interestingly, environmentally driven adult mortality can interact with sex and cause a sex gap in the lifespan, as recently demonstrated across cohorts of highly differentiated quality in two roe deer, *Capreolus capreolus*, populations [49]. When environmental conditions during the first months of life are harsh and thus associated with a high level of juvenile mortality, only high-quality females with substantial longevity prospects will survive to this selective sieve (i.e., a case of strong viability selection, see Box 1). The picture is different for males. Although males will suffer from the same level of juvenile mortality as females when poor environmental conditions are met during their first year, the likelihood of surviving from harsh environmental conditions early in life is independent of their quality, simply because the strength of the viability selection is lower on this sex. However, males carry the burden of a harsh early life by displaying a reduced lifespan compared to that of females [49].

### Male mortality due to male-male competition

The mammalian literature is full of case studies reporting that males display a higher level of environmentally driven adult mortality than in females (e.g., [50] in ungulates). Such a higher male mortality is often linked to the intensity of sexual selection that is much stronger in that sex [51, 52]. Originally introduced (1859) and developed (1871) by Charles Darwin, the concept of sexual selection defines an evolutionary process in which individuals from one sex compete for mating with individuals of the opposite sex [53, 54]. Because females generally produce a few energetically costly gametes during their reproductive life, while males repeatedly produce many small and motile gametes (i.e., anisogamy), females often constitute the limited sex (in terms of reproduction efficiency), and males face an intense sexual competition. The high level of sexual competition generally explains the “*live fast-die young”* life strategy observed in males [55], with males taking more risks of dying in their quest for mating opportunities. This is perfectly illustrated by the existence of fights that sometimes occur between conspecific males (e.g., [56]). Following Williams’ (1957) prediction, males should be the shorter-living sex because they suffer from a higher level of environmentally driven adult mortality, assuming that mortality is random rather than condition-dependent (which is, however, unlikely to be the case in natural populations of mammals, [57]).

### The cost of developing sexually dimorphic bodies

The stronger selection for gaining mating opportunities that act on males is also responsible for a myriad of physiological adaptations to sexual competition that might play an important role in shaping sex differences in mortality. In that context, sex-specific hormonal profiles constitute a striking example [10, 58]. For example, testosterone, which controls the development of the expression of many sexual traits in males (see below), is likely to have a negative impact on some aspects of biological performance, (e.g., immunocompetence, see [59]) and ultimately on male survival [52]. This is illustrated by the enhanced survival of castrated males observed in several mammals (e.g., [60] on Soay sheep *Ovis aries*), including humans. However, it is important to note that the sex gap observed in the lifespan of many species, again including humans, might be reinforced by possible survival benefits conveyed by the high level of estrogen exposure in females [58].

The hormonal regulation of the growth and maintenance of secondary sexual traits is thus likely to have profound deleterious consequences for male survival. Moreover, the substantial male allocation to specific sexual traits (e.g., large body mass or conspicuous ornaments in species such as beetles and bovids, [61, 62]) is expected to be energetically costly to grow and maintain throughout the entire life [63]. As predicted by current evolutionary theories of aging (e.g., the disposable soma theory, [64, 65]), the stronger reproductive expenditures by males during early years of life might be costly on the long run in terms of aging [66, 67]. In this context, sex differences in growth patterns provide a very striking example. In most sexually dimorphic and polygynous mammals, males grow faster and larger than females because attaining a large body rapidly mass can be advantageous to control access to reproduction [68]. However, rapid growth is associated with many physiological costs (e.g., decreased resistance to oxidative stress, [69]; increased oxidative damage and faster speed of telomere attrition, [70]; steeper rate of body mass decline with increasing age, [71]), possibly leading to a higher adult mortality [72]. Therefore, the strong allocation to growth by males in many species might result in decreased somatic maintenance and potentially of earlier or faster aging [65], which, with everything else being equal, increases the magnitude of the sex gap in aging in favor of females.

In male insects, the longevity costs of mating have been well-described thanks to experiments manipulating the male reproductive energy expenditures (see [52] for a review of empirical evidence). For example, in the social ant species *Cardiocondyla obscurior*, males experimentally assigned to a ‘high mating rate’ treatment showed a reduced lifespan by approximately 35% compared to that of males assigned to a ‘low mating rate’ treatment, with a result interpreted as a possible trade-off between reproductive energy expenditure (i.e., in spermatogenesis and/or courtship behaviour) and somatic maintenance [73]. Such mating costs have also been reported in wild populations of antler flies (*Protopiophila litigata*) in which long-lived males had a lower mating rate [74].

In vertebrates, the relationship between sexual selection and the sex gap of mortality has been principally approached using comparative analyses. In a recent study, Tidière et al. (2015) compiled 11 studies that investigated the role of sexual selection in shaping male and/or sex-specific longevity and aging patterns and highlighted the contrasted effects of the allocation to sexual selection depending on the taxa investigated or the metric considered [75]. For instance, in birds, where adult mortality is generally higher in females than in males [41], the intensity of male-male competition as assessed by the type of mating system, and the relative mass of the testes was positively associated with a male-biased mortality [41]. Sexual size dimorphism also seems to be associated with a male-biased mortality [40], although this relationship is sensitive to the species included in the analyses [40, 76]. So far, all avian studies have focused on the average estimates of adult mortality rather than on age-dependent aging metrics (e.g., rate of aging). In mammals, studies that investigated the relationships between sexual size dimorphism and male-biased mortality, longevity or aging metrics have been rather inconclusive [75]. However, in mammals the magnitude of the sex gap in mortality seems to be linked to the mating system. For example, in polygynous ruminants, between-sex differences in mortality are larger in polygynous than in monogamous ruminants for both longevity [35, 75] and the age at the onset of aging [75]. Interestingly, in ruminants living in zoos where mortality through direct male-male competition is virtually absent, this pattern holds, which suggests that the physiological/developmental costs of sexual dimorphism must contribute to those male-female differences in longevity [75].

### Sexual conflict and sex gap in mortality

Several authors have put forward the importance of sexual conflict in the evolution of sex differences in mortality patterns [77] because males and females maximize fitness in very different ways [78, 79]. The crucial role of sexual conflict has been recently discussed elsewhere (see [24, 52, 80] for extensive reviews of the putative role of both inter- and intra-locus conflicts in sex-specific aging). We provide here one salient example to illustrate their possible impact. Promiscuous females generally benefit from multiple mating events to maximize the fertilization success of their eggs [81, 82], but from the male perspective, the expected fitness can be strongly impaired by the consecutively high number of competitors. Several adaptations have evolved in males both to reduce female mating rates and to increase female allocation into current reproduction [80]. The harmful aspects of many of these traits have now been documented [79, 83]. For instance, experiments performed in *Drosophila melanogaster* have revealed that accessory gland proteins (e.g., the sex peptide, SP, [84]) contained in the male seminal fluid can stimulate the production of eggs by the female, while at the same, time reducing both her receptivity and lifespan [85, 86]. In the same way, in bean weevil (*Callosobruchus maculatus*) males bear sclerotized spines at the surface of their intromittent organs that damage the female reproductive tract during copulation and thereby diminish their lifespan [87]. This tactic can be highly beneficial to male fitness because injured females might both delay re-mating and increase their current allocation into reproduction [87]. While some vertebrate males might also potentially injure females during mating (e.g., through the presence of penile spines in some primates or rodents (e.g., [37, 88], the importance of sexual conflicts in terms of aging and longevity remain almost exclusively studied in insects through laboratory-based treatments ([80], but see also [89, 90]). This suggests that, at least in mammals, counter-adaptations to prevent female re-mating (e.g., copulatory plug) are not particularly deleterious to females. However, many more studies encompassing the diversity of reproductive tactics reported in male vertebrates are needed before drawing any firm conclusion about the absence of any role played by sexual conflict on the sex gap in the longevity and aging metrics in vertebrates.

Overall, the spate of the examples provided in this section, which encompass a broad range of studies based on laboratory animals and free-ranging populations, indisputably indicate that sex-specific reproductive strategies play an important role in shaping sex-specific mortality trajectories. However, much less is known about the underlying physiological and genetic mechanisms at the origin of these differences.

## Sex gap in aging and longevity due to asymmetries in genetic inheritance between sexes

### The mother’s curse

An aspect of the biology of males and females that could contribute to the sex gap in longevity and aging is the inheritance of some genetic factors that differ between sexes. This is the case with the mitochondrial genome, which is inherited maternally, and of the sex chromosomes that differ between sexes. In most species, mitochondria are transmitted through the female lineage and natural selection can only operate in that lineage. In particular, natural selection will be completely blind to deleterious mutations that (mainly) have a male-specific effect and those mutations can be passed through generations by females as if they were neutral mutations [91]. The accumulation of mainly male deleterious mutations in the mitochondrial genome could, in principle, explain why males age faster and die younger, which is called the “mother’s curse” hypothesis [24]. This hypothesis, however, predicts that longevity should systematically be female-biased (except for species with biparental transmission of mitochondria as for example some bivalves), which is not always what it is observed. Moreover, the mitochondrial genome includes very few genes in animals, and the potential to drive aging through the mother’s curse is probably small. Unfortunately, the mother’s curse has been studied in very few organisms and mainly in Drosophila. In Drosophila, some data support this theory [92]. In humans, some genetic diseases with a mitochondrial origin are known to affect males more than females. In one of them, Leber’s optic neuropathy, the mother’s curse has acted over 290 years and could be observed in a human population [93]. In plants, where mitochondria have a much larger genome size and gene content (e.g., [94]), and where chloroplasts also have a maternal inheritance, the mother’s curse has a much greater potential for explaining differences in longevity between males and females, which remain to be characterized.

### The effects of sex chromosomes

**Fig. 2:**
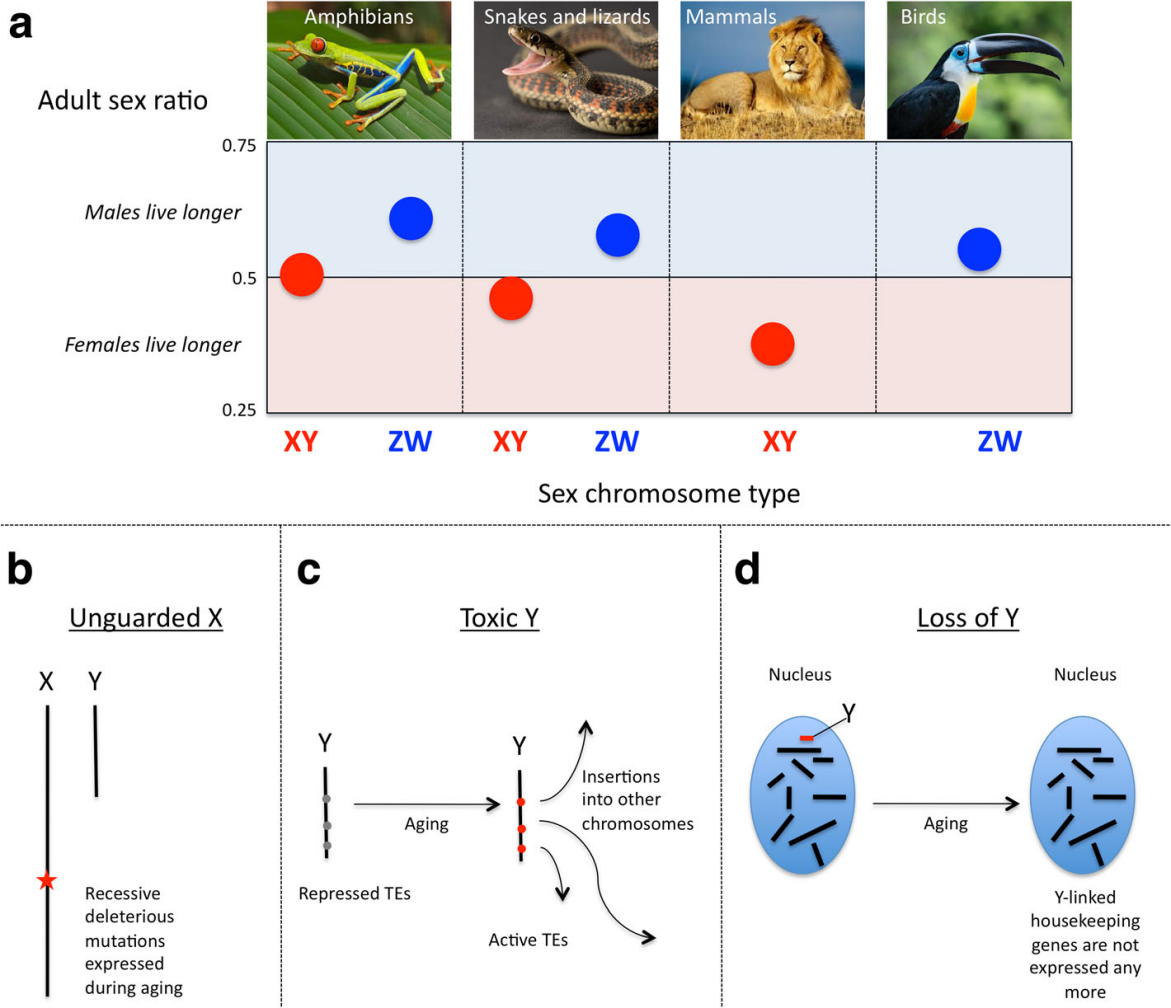
The contribution of sex chromosomes to sex-specific differences in longevity and possible mechanisms. **a** The relationship between either female-biased or male-biased adult sex ratios and the sex chromosome type in vertebrates (adapted from [99]). The mechanisms through which sex chromosomes can impact longevity: (**b**) the unguarded X effect, (**c**) the toxic Y effect and (**d**) the loss of Y chromosomes. See text for details

An obvious difference between men and women are the sex chromosomes, which could impact aging and longevity in a number of ways. A first obvious effect of having sex chromosomes is that males have one X and are hemizygous for that chromosome while females have two Xs. In women, however, X-chromosome inactivation (XCI) in the soma means that only one X is expressed in each cell. However, XCI is random in humans and other placentals, which means that, at the level of the tissue, on average, half of the cells will express the paternal X and the other half the maternal one, and functional diploidy is restored at the level of the tissue. This implies that if present in a male, a deleterious mutation on the X will always be expressed [95]. If present in a female, it will depend whether the mutation is recessive or dominant and whether that female is homozygous or heterozygous for this mutation. Indeed, it is known that most of the genetic diseases/conditions carried by the X chromosome have a much higher prevalence in men than in women (e.g., daltonism, hemophilia, Duchenne muscular dystrophy). This mechanism, called the “unguarded X”, could contribute to aging and longevity (Fig. 2b). Of course, the unguarded X effect depends on how well functional diploidy is restored in females in species with XCI. In humans, a skewed XCI is associated with faster aging and a shorter lifespan, and centenarian females tend to have a balanced XCI [15, 96, 97]. Why some individuals exhibit skewed XCI and not others remains to be understood.

A general prediction of the unguarded X is that, in XY systems, males should die faster. In some species (e.g., birds, butterflies), females are heterogametic (i.e., have different sex chromosomes); these systems are called ZW (females: ZW, males: ZZ). The W is equivalent to the Y and the Z to the X ([98]). In these systems, the unguarded Z effect should result in the opposite pattern: ZW females should die faster. Until recently, however, very little data was available and they tended to support the idea that sex chromosomes would not have a major role in sex-specific aging patterns. In particular, fragmented data on patterns of longevity and aging in birds suggested that females might outlive males in this taxon, contrary to what is expected with the unguarded Z. Sex chromosomes were thus disregarded in the literature about the sex gap in aging and longevity (e.g., [24]).

Some recent data have changed this view. Pipoly et al. (2015) have investigated the connection between sex chromosomes and aging/longevity by compiling data on adult sex ratios (ASRs) as a proxy for the sex gap in longevity and sex chromosome types (XY, ZW) for 344 species of tetrapods (including mammals, birds, lizards, crocodiles, snakes, amphibians), by far the largest dataset analyzed so far. They found a strong statistical association between the sex chromosome type and ASRs [99]. In the XY species, ASRs are female-biased, which suggests that males tend to die younger, whereas it is the opposite pattern in ZW species (Fig. 1a). Sex chromosomes are a widespread sex determination system in animals but also in other groups, such as plants and algae [100], and they could contribute to the sex gap in mortality in many groups and could be the major contributor in those where sexual dimorphism is not very strong, such as plants and algae [101]. Some other recent data suggests that the unguarded X/Z might be just one mechanism among several. In Drosophila, the Y chromosome, despite its very small gene content, has a major effect on the epigenetics of the other chromosomes [102]. Some recent data suggests that the epigenetics of the Y chromosome (i.e., DNA methylation, histone marks) change throughout life. In old male flies, Y chromatin is more open and transposable elements (TEs) tend to be de-repressed, which could result in those elements jumping around in the male genome, causing deleterious mutations and speeding up aging [103]. In line with this idea, another study in flies has shown that part of the variance in aging could be attributed to the genetic variance of the Y chromosome [104]. To further test the idea that the Y chromosome causes faster aging in males than in female flies, Brown and Bachtrog (2017) looked at aging and longevity in XXY females and monosomic X and XYY males, and confirmed that the Y increases aging in Drosophila [103]. This suggests that sex chromosomes may contribute to aging through a “toxic Y/W” effect because of particularly high transposable element content (Fig. 2b and Box 2).

Another possible mechanism through which sex chromosomes could affect longevity is cellular mosaicism (Fig. 2d). Cellular mosaicism is the presence of cells with different genotypes caused by somatic mutations. They include large-scale intra-chromosomic rearrangements and gain or loss of entire chromosomes. Cellular mosaicism is known to increase with age for all chromosomes in somatic tissues, but this increase is much higher for the sex chromosomes [105–109]. Large-scale somatic mutations, and, in particular, loss of the Y, increase in aging men and are associated with an increase in the risk of cancer [107]. Some external factors such as smoking are associated with increased rates of Y loss, and Y cellular mosaicism may contribute to an increased cancer risk with smoking [108]. Cellular mosaicism involving the X is also more frequent than that of autosomes [109]. The precise mechanism underlying these chromosome-specific differences in cellular mosaicism is not well understood, but in females, the inactive X is mostly affected [109]. In centenarian interphase cells, the loss of X in women (~ 22%) is higher than the loss of Y in men (~ 10%), which may suggest that the loss of the inactivated X has less consequence than the loss of the Y [110].

## Conclusions and perspectives for future research

Until recently, there was consensus in the literature that the main explanation for the sex gap in aging and longevity was a side-effect of sexual selection, sexual conflict and sexual dimorphism. Recent data suggests that sex chromosomes may contribute to those patterns. This is particularly true in species, such as monogamous birds, with no or very little sexual dimorphism, which display some sex gap in aging and longevity. The main approaches to studying the cost of developing sexual dimorphism on aging have involved comparative analysis and experimental evolution. Consequently, it is currently unclear what the underlying mechanisms are. To explore this further, it would be particularly interesting to study how the level of sexual dimorphism of an organ relates to its contribution to disease and death in humans.

Recent data suggests that sex chromosomes may affect aging and longevity, but this needs to be strengthened by more studies. In particular, the use of ASRs in the paper by Pipoly et al. (2015) is not optimal because ASRs can be affected by factors other than a sex gap in aging and longevity. Studies using other proxies for the sex gap in aging and longevity are probably needed. Comparative analyses could include species that have sex determination types other than sex chromosomes (e.g., environmental sex determination and monogenic or polygenic systems). No differences between males and females should be expected in those species if sex chromosomes are the main drivers in the studied taxon. Additionally, different mechanisms have been proposed for how sex chromosomes could affect aging: the unguarded X, the toxic Y or the loss of Y. Teasing apart these three mechanisms is not easy but needs to be done to understand the effect of sex chromosomes on aging. The unguarded X should be stronger in a highly polymorphic species and this could be tested. Polymorphisms could be changed experimentally, and one could see how this affects male/female viability, which has been done in the past in a few studies [111, 112]. The comparative approach could also be fruitful here, as unguarded X, toxic Y and the loss of Y are expected to have different effects on aging depending on the features of the sex chromosomes. For example, in X0 systems, no toxic Y or loss of Y effects are expected. In homomorphic systems where X and Y have very large pseudo-autosomal regions and only a small region of the X is hemizygous in males, no or little unguarded X effect is expected. The transposable element content or activity of the Y should be correlated with the speed of aging in males if the toxic Y hypothesis is correct.

Theories on the sex gap in aging and longevity are sometimes presented as opposing, but it is likely that sexual dimorphism, the mother’s curse and sex chromosomes all affect the aging and longevity of males and females with a species-specific relative contribution. Moreover, there might be some interaction between the different effects. For example, genes on sex chromosomes can be involved in sexual dimorphic traits [16, 113, 114]. To gain a general understanding of sex-specific differences in aging and longevity, we need to study these relative contributions and interactions. In humans, the available data suggests that sex hormones have a large effect on aging/longevity. Demographic data on people with sex chromosome aneuploidies suggest that sex chromosomes may play a role in human sex differences in aging as well. Compared to the normal population, individuals with an XXY karyotype (Klinefelter syndrome) have a 2-year reduction of longevity, whereas those with an XYY karyotype have a 10-year reduction [115], suggesting a strong toxic Y effect in humans. Manipulating sex chromosomes and gonadal sex could be a fruitful approach to tease apart the effect of sexual dimorphism and sex chromosome content on aging. A very promising model to do that involves the mouse, in which females and males with different sex chromosome content can be obtained using four-core gametes and the XY* systems [116].

In addition to the biological aspects discussed in this review, others might be relevant for the sex gap in aging and longevity, such as sex-specific differences in the gut microbiota [15, 117]. Lastly, understanding why males and females age differently and die at different ages is not only important for understanding the biology of sex differences but also for developing sex-adapted interventions on aging in humans.

## Box 1: The mortality key terms used in demography

*Annual adult mortality*: The rate of annual mortality observed in adult individuals from a population. It varies between 0 and 1 and is generally estimated as the ratio between the number of adults at time t+1 and the number of adults at time t (see Fig. 1).

*Life expectancy*: The number of time intervals that an individual of a given age is expected to live (based on the knowledge of the full age-specific survival curve). The life expectancy at birth is an often used metric at the population or species level. It corresponds to the *average lifespan* of this population or species.

*Lifespan*: The number of time intervals between the birth and the death of a given individual. Time is measured in years in most vertebrate populations. Individual lifespans can be averaged within populations (average population lifespan) or within species (average species lifespan). The maximum lifespan is an often used metric at the species level. It corresponds to the age at death reached by the longest-lived individuals. As such, the estimated maximum lifespan strongly depends on sample size (see Fig. 1).

*Longevity*: Synonym of lifespan.

*Onset of aging*: The age from which the annual adult mortality rate starts to increase. According to the predominant evolutionary theory of aging, the age of onset of aging corresponds to the age at first reproduction. However, recent empirical studies have revealed that the increase in the mortality rate with age generally occurs later. This term is often referred to as the “*onset of actuarial senescence*” (see Fig. 1).

*Rate of aging*: The rate of increase in the adult mortality rate with age. This term is often referred as the “*rate of actuarial senescence*” (see Fig. 1).

*Viability selection*: The process involves a non-random mortality that leads to a selective disappearance of individuals in relation to some phenotypic value. The most common situation corresponds to a selective disappearance of frailer individuals, which leads the proportion of robust individuals to increase with age. Viability selection can thus mask aging at the population level if not accounted for.

## Box 2: Transposable elements, epigenetics and aging

The process of aging has also been associated with a release of the epigenetic programme (such as DNA methylation or histone modifications), which modifies the gene regulation [122]. This has been described from humans to Drosophila, mainly in the somatic tissues, and has been proposed as one of the main reasons for the aging process [123–126]. A release of the epigenetic control will also have an impact on the control of the repeated sequences, such as TEs. TEs are repeated sequences that have been described in virtually all species, and can potentially move from one location to another in the genome. The consequences of this transposition are mainly deleterious, as the chromosomic rearrangements induced by ectopic recombination can be mediated by TEs. As a consequence, we expect TEs to accumulate in regions of low recombination, since natural selection will not be efficient to eliminate them. The Y chromosome, in which recombination is low, is thus prone to accumulating TEs. To silence TEs and avoid transposition, TEs are very often the target of epigenetic marks that will provide the necessary silencing and prevent too many deleterious effects of transposition. The idea that the heterochromatic regions, such as the Y chromosome, go through changes across age [127] and will be accompanied by the increased activity of TEs, with increasing rates of mutation, is very tempting. The importance of the Y chromosome in life history traits is not clearly understood, but several studies indicate that the influence of the Y chromosome may be quite important. More than 15 years ago, some Drosophila experiments showed that heat-induced male sterility was dependent on the Y chromosome [128]. The authors introduced wild Y chromosomes in two different backgrounds and specifically isolated the Y effects. More recent studies have revealed the implication of the Drosophila Y chromosome on the gene networks. Indeed, Y chromosome polymorphism affects the expression of genes that are located in the autosomes, some of them are related to male life history traits [102, 129–131]. A new study in *Drosophila melanogaster* suggests that epigenetic control of the Y is released with age, which results in TEs no longer being silenced by a repressive chromatin state, transposing all over the genome, provoking new mutations that reduce the fitness of individuals, and contribute to increasing cell death with age [103]. Life span surveys of XY, X0, XYY males and XX, XXY females suggest that this increase in transposition from the Y explains the aging differences between sexes in *D. melanogaster*. It would be interesting to carry out similar work in other model organisms, such as mice, for which we can obtain individuals with different numbers of Y chromosomes.

## Abbreviations

ASR: adult sex ratio
TE: transposable element
XCI: X-chromosome inactivation
